# Improving mental wellbeing of forensic psychiatric outpatients through the addition of an informal social network intervention to treatment as usual: a randomized controlled trial

**DOI:** 10.1186/s12888-020-02819-2

**Published:** 2020-08-25

**Authors:** L. T. A. Swinkels, T. M. van der Pol, A. Popma, J. F. ter Harmsel, J. J. M. Dekker

**Affiliations:** 1 Inforsa Forensic Mental Health Care, Vlaardingenlaan 5, 1059 GL Amsterdam, the Netherlands; 2 Department of Child and Adolescent Psychiatry, Amsterdam University Medical Center, Meibergdreef 5, 1105 AZ Amsterdam, the Netherlands; 3 Department of Research, Arkin Mental Health Care, Klaprozenweg 111, 1033 NN Amsterdam, the Netherlands; 4 Department of Clinical Psychology, VU University, Klaprozenweg 111, 1033 NN Amsterdam, the Netherlands

**Keywords:** Randomized controlled trial, Forensic psychiatry, Social network intervention, Mental wellbeing, Social network, Social support, Criminal recidivism

## Abstract

**Background:**

Forensic psychiatric patients often suffer from a multitude of severe psychiatric and social problems. Meanwhile multimodal evidence-based interventions are scarce and treatment effectiveness is in need of improvement. The main goal of forensic psychiatric treatment is to address psychiatric and social factors and thereby mitigate criminal behaviour. Notably, a supportive social network is an important protective factor for criminal behaviour. As such, improving a poor social network may decrease the risk of criminal recidivism. This study aims to examine the effectiveness of the addition of an informal social network intervention (FNC) to treatment as usual (TAU) among forensic psychiatric outpatients.

**Methods:**

In a mono-center randomized controlled clinical trial with two parallel groups, forensic psychiatric outpatients with social network-related problems (*N* = 105) will be allocated to either TAU + FNC or TAU alone. The informal social network intervention consists of a 12-month coaching intervention, performed by the forensic network coach (a volunteer trained by an informal care institute). Assessments will be conducted at baseline and 3 months, 6 months, 9 months, 12 months, and 18 months after baseline. The primary outcome variable is mental wellbeing. Psychiatric functioning, criminal recidivism, substance abuse, quality of life, social network, social support, loneliness and self-sufficiency are included as secondary outcomes. A variety of potential mediators and moderators of effectiveness will be explored. Additionally, a qualitative evaluation of effectiveness will be performed.

**Discussion:**

This study will contribute to the existing literature of forensic treatment effectiveness as it is the first RCT examining the effectiveness of adding a social network intervention in a forensic outpatient population. If effectiveness is shown, forensic mental health care could be optimized by collaborating with informal care or community initiatives aimed at improving a positive social network. In addition, results will provide insight regarding mediators and moderators of treatment effectiveness.

**Trial registration:**

This study is registered at the Netherlands Trial Register (NTR7163). Date of registration: 16 april 2018.

## Background

Forensic psychiatric patients often suffer from severe mental disorders, addictions and intellectual disabilities, combined with delinquent behavior [1–4]. In addition, they are confronted with multiple social problems, including housing problems, unemployment, financial problems and social network-related problems [5–8]. Given the complexity of these problems, forensic psychiatric patients are in urgent need of effective treatment. The main goal of forensic psychiatric treatment is to reduce the risk of criminal recidivism. Therefore, forensic psychiatric treatment should be multimodal and personalized; focused on improvement of the social context of the individual patient and multiple mental health problems, related to their delinquent behaviour [9, 10].

Forensic psychiatric treatments that comply with the Risk-Need-Responsivity (RNR) principles are found to be most effective [11]. The RNR-model is used worldwide for the indication and execution of forensic treatments [12, 13]. This model emphasizes that treatment is effective if: 1) the level of treatment intensity is matched to the risk level of criminal recidivism - Risk principle, 2) the criminogenic needs related to criminal recidivism are addressed - Need principle, and 3) the type of intervention is matched to the abilities and skills of the delinquent - Responsivity principle [14]. Forensic assertive community treatment (forensic ACT) and forensic flexible (or function) assertive community treatment (forensic FACT), a Dutch adaptation of forensic ACT [15], are examples of broadly applied multimodal treatment models for forensic psychiatric outpatients [15–17]. In order to prevent criminal recidivism, forensic (F) ACT incorporates the RNR-principles by focusing on multiple individual and systemic risk factors (e.g. financial or cultural barriers, a lack of services) as well as patient’s nonadherence [18–20]. However, there is much variation between individual forensic (F) ACT treatment trajectories and core elements are not well defined [21]. Furthermore, many previous effect studies showed methodological limitations; results from randomized controlled trials (RCTs) are scarce [22]. Overall, the effectiveness of outpatient forensic interventions is limited. A meta-analysis showed positive effects – statistical significant declines of criminal recidivism in the experimental conditions – in only a minority (32.3%) of the forensic interventions [23]. Nevertheless, forensic (F) ACT is a well described and promising multimodal treatment model that meets the complex needs of forensic patients in clinical practice. In order to further improve treatment effectiveness for this vulnerable group, working mechanisms of the current (multimodal) forensic treatments models should be studied extensively. Hence, more RCTs are necessary to develop specific evidence-based interventions [24].

Social network-related problems of forensic psychiatric populations are considered one of the important targets within treatment [25–27]. A substantial part of the social network of forensic patients is engaged in criminal activities or consists of network members with psychiatric problems and substance (ab) use [5, 28]. Also enhanced levels of loneliness are observed in prison and forensic inpatient populations [29, 30]. Both the absence of social support and the presence of a criminal social network of family and friends has been related to increased criminal behavior [31–33]. Indeed, a supportive social network - a network of personal contacts that contribute to a social identity, new contacts and provide emotional, instrumental and material support [34] - has been postulated as an important protective factor against criminal recidivism [35]. As such, strong social support, stable contact with positive peers and social ties with prosocial adults are related to less criminal recidivism rates [35–37].

Until now, systematic reviews showed that social network interventions lead to health behavior improvements in general populations [38]. Among psychiatric populations, social network interventions focused on network-related problems and family relationships, can lead to improved mental wellbeing and a better use of mental health services [39, 40]. Despite these results and the positive influence of social support on criminal recidivism, social support interventions are not actively applied (in addition to treatment as usual) in forensic populations [26, 41]. Moreover, RCTs and other evaluative studies that examine the effectiveness of social network interventions in forensic psychiatric populations are scarce. One pilot RCT that compared a short group social support intervention with a general re-entry service among a small sample of recently released prisoners, found no statistically significant group effects for social support, cognitions, substance use and recidivism [42]. However, larger sample sizes are recommended to reliably investigate the effectiveness of the intervention. Furthermore, the intervention comprised of a short intervention (i.e. 10 week training), while researchers emphasize that social support interventions for forensic psychiatric patients should be long lasting in order to find significant effects [27, 42]. An evaluative study of a social support intervention among mentally ill prisoners during and after incarceration found a significant positive association with quality of life, but no significant association with criminal recidivism or psychiatric hospitalisation [43]. The generalizability of these results is limited due to a small sample size and low treatment adherence rates. Other researchers found some positive results - improved criminal recidivism outcomes - in a group of former prisoners that received social support by community volunteers [41]. The addition of an informal or volunteer-based social network intervention could be promising, since social networks of forensic patients are often overrepresented by formal or professional health caregivers [28]. Improvement of supportive social networks during treatment of forensic psychiatric patients with social network-related problems might reduce criminal recidivism directly or indirectly, for example by enhancing mental wellbeing which may lead to reduced mental health problems and criminal recidivism. More RCTs with a sufficient sample size are needed to examine the effectiveness and working mechanisms of social network interventions in forensic psychiatric populations.

Therefore, in the present study, a randomized parallel-group RCT will be conducted to examine the addition of Forensic Network Coaching (FNC) - an informal social network intervention - to (a multimodal) treatment as usual (TAU) among forensic psychiatric outpatients. The aim of FNC is to: 1) improve (the quality of) social networks, 2) social support, and 3) social participation. To provide an accessible social network intervention and stimulate patients’ adherence to the intervention, FNC will be tailored to the individual needs. To promote effectiveness, FNC is available for a prolonged period of maximum 12 months to promote effectiveness. To our knowledge, this is the first RCT that examines the additional effectiveness of an informal social network intervention within forensic clinical practice. Hence, this study will contribute to generalizable knowledge about the effectiveness of (specific) interventions for forensic psychiatric populations.

### Research aims

The first aim of this study is to examine the effectiveness of the addition of FNC to treatment as usual (TAU) on the mental wellbeing among forensic psychiatric outpatients with social network problems and limited social participation. We expect significantly enhanced mental wellbeing rates in FNC as compared to TAU alone, by improving social networks, social support and social participation. The second aim is to examine the effectiveness of the addition of FNC on psychiatric functioning, criminal recidivism, addiction and other secondary outcomes (quality of life, social network, social support, loneliness, self-sufficiency). We expect significant improvements on psychiatric functioning, criminal recidivism and addiction, as well as on other secondary outcomes, in the FNC condition as compared to TAU. The third aim of this study is to gain insight into the development of the primary and secondary outcomes over time. To enhance knowledge regarding the underlying mechanisms, a variety of potential mediators and moderators will be explored.

## Methods

### Design

**Fig. 1:**
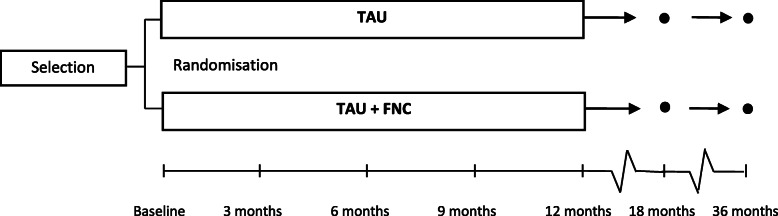
Overview of the trial design

A mono-center open label RCT with a two parallel group design will be conducted. A total of 105 eligible participants will be randomly allocated to either TAU with the addition of FNC or TAU alone, after the first baseline assessment. All participants receive treatment as usual within a forensic mental health care institute at the time of the baseline assessment. Participants in the FNC condition are offered an additional intervention – Forensic Network Coaching – consisting of two-monthly appointments with a forensic network coach. Assessments will take place before randomization at baseline and every 3 months after baseline assessment until the final assessment at 12 months of treatment. The follow-up assessment will be conducted at 18 months after baseline assessment. Number of crime occurrences and recidivism rates, will be determined at 12 months and 36 months after baseline assessment*.* The effectiveness of FNC will be examined at 12 and 18 months after baseline assessment. Approval for the study protocol was given by the Medical Ethics Committee of the VU University Medical Center (registration-number NL60308.029.17). The study is registered at the Netherlands Trial Register, part of the Dutch Cochrane Center (NTR7163). Figure 1 provides an overview of the trial design.

### Participants

This study is conducted at the outpatient care site of Inforsa Forensic Mental Health Care, specialist in forensic psychiatric care for (young) adults, located in Amsterdam. Inforsa consists of three different forensic outpatient care sites: 1) forensic flexible assertive community treatment (forensic FACT) for adults, 2) forensic FACT for youth and young adolescents, and 3) forensic outpatient clinic. The overall patient population of Inforsa is characterized by complex addiction, psychiatric and personality disorders in combination with delinquent behavior. The type of crimes committed by patients are diverse, most common offences are violence or theft. Treatment is a mandatory part of a conditional sentence, for 87% of the patients. The majority of the patients (69%) suffers from psychiatric or personality disorders in combination with addiction - double diagnoses. On top of that, at least 11% of the patients suffers from intellectual disabilities - triple diagnosis. Most patients have social problems such as housing problems, work problems and financial problems.

Our goal is to include 105 (young) adult forensic outpatients with limited social networks and social participation. Inclusion criteria are: 1) the patient is at least 3 months in treatment at Inforsa and capable of accomplishing appointments according to the primary clinician, 2) the patient is diagnosed with addiction, psychiatric or personality disorder according to the Diagnostic and Statistical Manual of Mental Disorders (Fourth edition, Text Revision and Fifth edition; DSM-IV-TR/5), 3) the patient is aged 16 years or older, 4) the patient is indicated as limited self-sufficient regarding social participation and social network as measured with corresponding items on the Self-Sufficiency Matrix (SSM) [44, 45], and 5) the patient is not completely satisfied with social relationships and the social support as measured with the Manchester Short Assessment of Quality of Life (MANSA) [46]. Exclusion criteria are: 1) acute psychotic symptoms according to the clinician or DSM-IV-TR/5 criteria as measured with the MINI International Neuropsychiatric Interview-plus (version 5.0; MINI-plus) [47], 2) current high risk for suicide requiring immediate intervention according to the clinician and/or DSM-IV-TR/5 criteria as measured with the MINI-plus, 3) severe addiction problems as measured with the Health of the Nations Outcome Scales (HoNOS) [48] requiring immediate intervention or hospitalization, 4) a current high risk for severe aggression towards clinicians or others as measured with the HoNOS, and 5) patient is included in another scientific research project conducted at Inforsa.

### Sample size

As our primary outcome variable is the total mean score on a self-report questionnaire - Mental Health Continuum - Short Form (MHC-SF) [49] - which measures mental well-being using Likert scaling, we consider this outcome as a continuous variable. Normality of the distribution will be checked after data collection using optimal scaling or scatter plotting. When the assumption of normality is violated, resampling methods will be used to transform the data into a normal distribution.

An accurate sample size estimation is challenging since no other studies have been performed with a comparative experimental design and intervention within the same population. Former studies evaluating mentoring programs show small to medium effect sizes [50–53], whereas studies investigating psychological, educational and behavioral treatments show larger mean effect sizes [54]. Effect sizes of life coaching performed in a nonclinical sample show medium to large effect sizes [55]. Given the foregoing, we expect a small to medium improvement of mental well-being in the FNC condition compared to TAU. We consider this a realistic and clinically meaningful effect. Our first power calculations (conducted with G*Power) indicated that 150 participants are needed (75 per arm) to detect a small-to-medium effect size (Cohen’s *f* = .20), given an alpha of 0.05 (two-tailed) and a power of 0.80. Since this power calculation did not account for the repeated measures design of the study, more modern power calculations were performed (conducted with STATA) to calculate the required sample size. To detect a small-to-medium effect size (Cohen’s *f* = 0.20) in a pairwise comparison of pre-post change between two active treatment arms, with a power of 0.80 and a within-person correlation coefficient of 0.50, 68 participants are needed in this study (34 per arm). We expect 30% of the participants as early drop-outs of the study [56], therefore a total of 105 participants will be included in the study.

### Procedure

#### Recruitment and consent

Patients will be recruited at all outpatient sites of Inforsa Forensic Mental Health Care, a department of Arkin Mental Health Care, a large mental health care institute in Amsterdam, the Netherlands. Recruitment is planned for 2 years. Patients will be screened for eligibility by a clinician and researcher during treatment. Researchers will inform and stimulate clinicians to refer patients by participating in team meetings, spreading information flyers, reminding clinicians, spreading newsletters and monthly presentations. When eligibility is assumed, the clinician will introduce the study to the patient to check whether the patient is interested in participating. After a patient expresses interest, more extensive screening between the clinician and researcher will take place.

Subsequently, the first recruitment appointment will be scheduled with eligible patients who agreed to be approached by a researcher. During the first appointment the patient will be screened for eligibility by a researcher. Furthermore, patients’ motivation for participation will be assessed. All eligible patients receive a written study information letter. The content of the study information letter will also be explained by a researcher. Depending on the preferences of the patient, the second appointment for the screening interview, informed consent and baseline assessment will be planned. The second appointment will take place at least a week after the first face-to-face appointment so enough time for consideration is provided. In addition, the researcher asks whether the patient agrees to be approached by a research assistant by phone, in order to check agreement with participation, to answer questions and to plan appointments for follow-up assessment.

During the second appointment, informed consent will be signed by all patients. After informed consent, baseline assessment will take place in which all in- and exclusion criteria are checked again by a researcher. If necessary - to decrease the burden for participants - baseline assessment will be divided into multiple appointments. The baseline assessment should preferably be completed within 1 month after informed consent.

#### Randomization and procedure

Independent research assistants and research associates - not familiar with the patient or responsible for any kind of mental health care - of the research department of Inforsa are involved in the recruitment procedure, randomization, data collection and data management of this study. Randomization will be performed at an individual level, stratified by forensic outpatient care site. We aim to carry out randomisation after completion of (the first half of) the baseline assessment. A computer-generated block randomization schedule provided by Castor Electronic Data Capture (EDC) is used. The allocation ratio will be 1:1 to ensure that an equal number of patients will be allocated to either TAU or FNC. Researchers will not have access to the randomization schedule and will be blind to block size and order. Blinding for treatment after randomization is not possible, as the content of treatment will be clear to both patients and therapists. Blinding researchers is not feasible due to different assessments of the conditions and required coordinating activities - referring patients to FNC and monitoring - of researchers during the FNC intervention.

The experimental intervention starts preferably within 2 months after completion of the baseline assessment and ends 12 months after baseline assessment. During the intervention, patients in both FNC and TAU condition will be assessed at 3, 6 and 9 months after baseline assessment. At the end of treatment (12 months) and follow-up (18 months) additional questionnaires will be administered. Figure 1 provides an overview of the trial design. Two supplemental measures, the Work Alliance Questionnaire (WAV-12) and a qualitative interview, will only be assessed in the experimental group. Table 1 provides an overview of all instruments and the corresponding time of assessment.

**Table 1:** Overview of outcome measures, instruments and time of assessment from baseline in months

Outcome	Concept	Instrument	baseline 0	3	6	9	end 12	follow-up 18	36
**Primary**	Mental wellbeing	MHC-SF	•	•	•	•	•	•	
**Secondary**	Psychiatric functioning^a^	HoNOS	•	•	•	•	•	•	
	Criminal recidivism	SRD	•				•	•	
		IPOL					•		•
		JDS					•		•
	Substance abuse	MATE 2.1	•	•	•	•	•	•	
	Quality of life	MANSA	•	•	•	•	•	•	
	Social network	NGI-method	•				•	•	
	Social support	SSL	•				•	•	
	Loneliness	Loneliness Scale	•				•	•	
	Self-sufficiency	SSM-D	•	•	•	•	•	•	
**Mediators / Moderators**	Diagnostic classifications	DSM-IV-TR/5	•						
		MINI-plus	•						
	Life trauma events	CTQ-SF	•						
	Attachment style	ASQ	•				•		
	Demographic characteristics	Self-developed	•				•	•	
	Working alliance^b^	WAQ-12		•	•	•	•		
	Intellectual disabilities	SCIL	•						
	Cognitive distortions	HIT-Q	•						
	Program integrity^c^	Self-developed					•		
**Other variables**	Qualitative interview of experiences ^b^	Self-developed					•		

^a^ this outcome measure will be scored by a researcher based on interviews, questionnaires and observations during assessments and information from patient files

^b^ this interview will be assessed for participants and forensic network coaches in the experimental group

^c^ this outcome measure will only be assessed for forensic network coaches in the experimental group

Forensic network coaches will receive an e-mail with a request to fill in one online questionnaire at 3, 6, 9 and 12 months after baseline assessment. In addition, after termination of the experimental intervention at 12 months, forensic network coaches will be interviewed face-to-face to measure program integrity and coaching experiences. Audio recordings will be made of every qualitative interview with both patients and coaches. Besides, researchers fill in questionnaires about patients in both conditions, at 3, 6, 9, 12 and 18 months after baseline, based on observations during assessment and information from the electronic patient file. Registered criminal recidivism data at 12 and 36 months after baseline will be requested by a researcher after termination of the study.

All assessments with patients - interviews and questionnaires - will be conducted face-to-face, using both paper and pencil and online questionnaires using Castor EDC. The research assistant will help participants with possible problems and difficulties during assessments. The data collection process will be carefully monitored. All patients and forensic network coaches will be frequently motivated by research associates to complete the assessments. If necessary, patients and coaches will be reminded via the phone or e-mail. If necessary, the assessments can also be completed by phone. All patients will be compensated with a gift card of €10,- after each assessment at 3, 6, 9 and 12 months. After completion of the last assessment, 18 months after baseline, patients receive a gift card of €25,-.

All collected data from interviews and questionnaires will be entered and stored online and in the onsite database. To promote data quality researchers will be trained in the study protocol and adequate administration of assessments. Weekly supervision sessions and team training meetings will be used to evaluate assessments. All data will be checked on missing data or specific errors or contradictions in data. Most of the time assessments will be filled in by patients on paper, therefore double data entry procedures will be performed to prevent defaults in data entry. Data entry and encoding by research associates will be consequently checked by a second associate. Besides, independent random data checks will be conducted on entry and encoding defaults.

Participants may withdraw from the study or experimental intervention for any reason at any time. Discontinuation of treatment as usual or early discontinuation of the experimental intervention is not a reason for withdrawal from the study. In case of early discontinuation of the intervention patients will be motivated for participation during the following research assessments.

### Interventions

#### Treatment as usual (TAU)

The vast majority of potential participants receives forensic FACT which can include specific therapeutic interventions such as social work, psychotherapy (e.g. CBT, EMDR) and pharmacotherapy. We will include patients at the forensic outpatient care site not receiving forensic FACT as well if social network-related problems are present and social participation is limited. All forensic outpatient care sites include psychotherapies and treatment models that are part of the treatment guidelines for forensic outpatients.

In short, forensic FACT is a widely used treatment model developed for forensic outpatients with severe psychiatric disorders, combined with multiple social problems [19]. The primary goal of forensic FACT is to enhance (mental) wellbeing as well as to reduce the risk of criminal recidivism. Forensic FACT teams consist of different health care professionals such as psychiatrists, nurses specialized in psychiatric problems, psychologists, job coaches and social workers. Treatment is delivered within the community of the patients. In general, individual face-to-face appointments between professionals and patients take place weekly at the institution or at a patients’ home. The intensity of the treatment is customized to the patient’s needs. If necessary, for example in case of (the prevention of) a crisis, the frequency of appointments can be increased and more professionals can be involved. Furthermore, CBT or psychotherapeutic interventions are indicated and performed by psychologists based on the patients’ needs and their individual risk assessment of criminal recidivism. Examples of common interventions are aggression regulation therapy [57], CBT for alcohol- or drug-use disorders [58], community reinforcement approach [59], delict analysis [60] and motivational interviewing [61]. CBT interventions and psychotherapy consist of weekly face-to-face appointments at the participating site. Besides, interventions may also be offered online or blended.

Care professionals of all patients included in the study (TAU and FNC) will be stimulated by the research team to focus on improvement of the social network during treatment. After baseline and follow-up assessments professionals will receive an e-mail with information about a brief social network intervention and to inform them about their patient’s participation. TAU might be discontinued or finished during the course of the study, therefore the type and duration of treatment will be monitored. No treatments are withheld from patients in TAU except Forensic Network Coaching.

#### Forensic network coaching (FNC)

In the experimental group a social network intervention - Forensic Network Coaching (FNC) - will be added to TAU during a period of maximum 12 months. FNC is an informal coaching or mentoring program that aims to improve (the quality of) social networks, social support and social participation. A carefully selected volunteer - a forensic network coach - is trained to conduct a structured intervention ‘Of course, a network coach!’ [62].

The intervention ‘Of course, a network coach!’ is included in an extensive list of effective social interventions by Movisie, a Dutch knowledge institute that focuses on social issues. Although empirical research has not been conducted, promising results have been published. A qualitative study shows that participants gained self-confidence, energy and social skills after completing the coaching intervention [63]. Furthermore, the intervention is based on the TO GROW (Goal Reality Options Will) model of coaching [64] and on Solution Focused (Brief) Therapy (SFT) [65, 66]. SFT is known to be an effective intervention in reducing psychiatric problems [67–69], improving mental wellbeing and social- and problem-solving skills [55, 70–73].

The intervention consists of ten steps: step 1 to 3, the Orientation phase, focuses on exploring patients’ wishes regarding the social network and determining goals. Subsequently, in the Thinking phase, step 4 and 5, both coach and patient will explore possible ways to improve or rebuild the social network. Lastly, in the Action phase, step 7 to 10, formulated plans will be executed. Every plan involves three components: 1) explanation for the coach, 2) a patient worksheet, 3) a practical and theoretical form for the coach. However, due to the characteristics of the forensic population - motivational challenges, disabilities and complex psychiatric problems - this intervention will merely be used as a theoretical guideline and tool for coaches while working on the improvement of the social network of participant. The intervention will be adjusted to the needs, pace and possibilities of the participant.

During FNC, patients are matched to a forensic network coach based on their personal preferences, characteristics and interests, after an intake appointment with De Regenboog Groep.^1^ Prior to the intake, necessary information regarding the social situation of the patient is provided by a member of the research team – a procedure for which written consent is given. If matching is successful - both coach and patient agree with the match - coaching appointments take place once every 14 days. Between appointments, coaches and patients may engage in ear-to-ear or screen-to-screen contact. Within the first three to 6 months of FNC, coaches are stimulated to focus on enhancing motivation and building a working alliance with their patient. The following months will be used to draft personal goals and gaining new social experiences. Forensic network coaches visit patients in their own social environment. Besides, coaches and patients are stimulated to participate in new social activities. Evaluation of FNC takes place every 3 months and after completion of FNC, at 12 months of coaching. Both coaches and participants can decide to continue the contact after completion of FNC.

#### Forensic network coaches

The forensic network coach is a carefully selected and trained volunteer who can be a role model and a supportive network member for the patient. The relationship between the coach and patient is called informal, meaning that coaches are non-professional volunteers that do not receive payments for their services. Furthermore, the relationship between a coach and patient is based on equivalence, confidence and liberty. Coaches are not involved in the treatment as usual.

Forensic network coaches will be provided by De Regenboog Groep, experienced in delivering coaching programs. De Regenboog Groep is responsible for the selection, training, matching and supervision of forensic network coaches. All coaches receive training in basic coaching skills, the social network intervention and on how to provide informal care within a complex forensic population. Coaches receive a manual with a worksheet of the intervention ‘Of course, a network coach!’. In addition, to ensure treatment fidelity, coaches will receive regular group supervision meetings. All coaches will be monitored by a professional of De Regenboog Groep during the coaching phase.

### Primary outcome measure

#### Mental wellbeing

The Dutch version of the Mental Health Continuum - Short Form (MHC-SF) will be administered to measure the current psychological, emotional and social wellbeing during the last month [49, 74]. The MHC-SF is a 14-item self-report questionnaire that consists of a 5-point Likert scale. The psychometric features of the MHC-SF are good. There is evidence for a high internal and moderate test-retest reliability and good convergent and discriminant validity [74]. The mean score of the psychological, emotional and social wellbeing scales - the (positive) mental wellbeing score – will be the primary outcome of this study. Assessment of the primary outcome variable will be conducted at baseline and 3, 6, 9, 12 and 18 months after baseline. The mean difference of the mental wellbeing score between groups over time - from baseline to assessment at 12 months - will be chosen as the main outcome measure of this study. Table 1 provides an overview of all instruments and the corresponding time of assessment.

### Key secondary outcome measures

#### Psychiatric functioning

The Health of the Nations Outcome Scales (HoNOS) is a 12-item, 4-point Likert-type scale, for the assessment of general and psychiatric functioning of people with severe mental problems [48, 75, 76]. The HoNOS consists of four subscales: behavioral problems, deficiencies, symptomatology and social problems. The HoNOS is widely used by clinicians. It is a valid instrument for the assessment of the effectiveness of mental health services, with good overall interrater and test-retest reliability, criterion validity and concurrent validity [48, 77]. The Dutch version of the HoNOS will be completed by a researcher at baseline and follow-up assessments at 3, 6, 9, 12 and 18 months after baseline, based on observations during assessment and information from the electronic patient file.

In addition, the number of renewed hospitalizations will be monitored throughout the study. We will ask participants whether and how frequently they have been hospitalized at all assessments. The number of hospitalizations during the study will also be gathered from electronic patient files.

#### Criminal recidivism

Self-reported criminal recidivism will be measured with 29 items of the Self-Reported Delinquency scale (SRD) [78]. Participants are asked if they have committed a list of 29 specific criminal activities (yes/no). Furthermore, they are asked to make an estimation about the number of total criminal activities throughout their lives and in the past 6 months. The criminal activities vary from minor to rare and serious offences. A total delinquency score can be obtained and the total delinquency score can also be divided into five subscale scores: public order offenses, property crimes, violent crimes, drug-related crimes and owning illegal weapons. Self-report approaches of criminal recidivism have demonstrated acceptable reliability and validity [79]. The SRD will be measured at baseline and 12 and 18 months after baseline assessment.

Additionally, information about the type, number, severity and time of arrests and offenses will be collected from the International Police Information Service (IPOL) and the Justice Documentation System (JDS) of the Dutch Ministry of Safety and Justice [80]. We will collect data from these records at 12 and 36 months after baseline assessment. Furthermore, the number of renewed incarcerations will be monitored throughout the study. We will ask participants whether and how frequently they have been incarcerated at all assessments. The number of incarcerations during the study will also be gathered from electronic patient files.

### Other secondary outcome measures

#### Substance abuse

Two chapters of the Measurement in the Addictions for Triage and Evaluation (MATE 2.1) [81] will be used for the assessment of alcohol and drug use as well as alcohol and drug abuse or dependency according to the DSM-IV-TR. Chapter one of the MATE 2.1 will be assessed at baseline and 3, 6, 9, 12 and 18 months after baseline, to measure the prevalence, frequency and average amount of an extensive list of substances (e.g. nicotine, alcohol, tranquillizers, drugs) in the last 30 days. In addition, the onset and duration of frequent substance use and the primary, secondary and tertiary problematic substances can be determined. Chapter four of the MATE 2.1 consists of 11 questions (yes/no) that can be used to determine alcohol and drug abuse or dependency in the past 12 months. Therefore, chapter four will be assessed at baseline, and 12 and 18 months after baseline. The MATE 2.1 has acceptable validity and reliability scores [82].

#### Quality of life

Quality of life will be measured with the Dutch version of the Manchester Short Assessment of Quality of Life (MANSA), a 16-item 7-point Likert-type self-report questionnaire [46]. The MANSA consist of 12 Likert-type questions that are related to important domains of life such as physical health, mental health, safety, social relationships, leisure activities, work and finance. Additionally, four yes/no-questions regarding victimization, delinquent behavior and friendships are included. The questionnaire demonstrated adequate psychometric properties [46]. The MANSA will be measured at baseline, and 3, 6, 9, 12 and 18 months after baseline assessment.

#### Social network

The size and quality of the social support network is measured with a self-developed interview based on the Modified Multiple Generator (MMG) [83] and Name Generator/Interpreter method (NGI) [84]. The NGI method is widely used in sociological research to investigate the size and quality of different type of social networks and changes in social networks [83, 85]. The MMG approach is a time-saving alternative of the NGI method, and therefore more appropriate for the current study. Two widely used name generator questions (“who are the people with whom you discuss matters important to you” and “who are the people you really enjoy socializing with”) are administered to determine the size of the social support network. If participants report more than five names, they will be asked to mention the five most important network members. Additionally, name interpreter questions are administered to measure the demographic characteristics of network members, the quality of the relationships and contact frequencies. Even though content validity of the MMG approach is less strong, when compared with the full NGI method, the MMG is found to be an acceptable alternative [83]. The instrument will be administered at baseline and 12 and 18 months after baseline.

#### Social support

Social support is measured with the Social Support List (SSL), a 41-item 4-point Likert-type self-report questionnaire [86, 87], at baseline and 12 and 18 months after baseline. The SSL consist of 34 items that refer to supportive interactions and seven items that refer to negative interactions. Positive interactions can be divided into six subscales: 1) everyday emotional support, 2) emotional support in case of problems, 3) support from the expression of validation, 4) instrumental support, 6) social companionship, and 6) informative and supportive feedback (regarding participants’ behavior). Furthermore, negative interactions can be divided into three subscales: 1) everyday support, 2) support in case of problems, and 3) support from the expression of validation. The SSL has acceptable validity and reliability scores [87].

#### Loneliness

Loneliness will be measured with the Loneliness Scale, a 11-item 5-point Likert-type self-report questionnaire [88, 89], at baseline and 12 and 18 months after baseline. The Loneliness Scale scores can be divided into emotional loneliness and social loneliness. The questionnaire demonstrated good psychometric properties [89, 90].

#### Self-sufficiency

Self-sufficiency will be measured with the Dutch version of the Self-Sufficiency Matrix (SSM-D) at baseline, and 3, 6, 9, 12 and 18 months after baseline assessment. The SSM-D is a 13-item 5-point Likert-type assessment tool of treatment outcomes [44, 45, 91, 92]. Self-sufficiency on 11 important domains of life, such as physical health, mental health, addiction, income, housing, social network, social participation and justice, are measured. The SSM-D is validated for the Dutch population [91] and demonstrated adequate psychometric properties [93, 94].

### Potential mediators and moderators

#### Diagnostic classifications

The presence of current psychiatric disorders based on the Diagnostic and Statistical Manual of Mental Disorders (Fourth edition, Text Revision and Fifth edition; DSM-IV-TR/5) and the International Classification of Diseases (Tenth revision; ICD-10) will be assessed with the MINI International Neuropsychiatric Interview-plus (MINI-plus; version 5.0) [47, 95]. The MINI-plus is a structured, clinician-administered, diagnostic instrument that consists of several sections (A-Z) that address different DSM-IV-TR and ICD-10 classifications. The MINI-plus is found to be a reliable and validated structured diagnostic instrument [95]. Section A-J, M, Q and W of the Dutch version of the MINI-plus [96] will be administered by the researchers at baseline to assess the presence of depressive disorders, suicidality, bipolar disorders, anxiety disorders, posttraumatic stress disorder, psychotic disorders, antisocial personality disorder and attention deficit hyperactivity disorder, respectively. In addition, DSM-IV-TR/5 classifications will be collected from electronic patient files at baseline.

#### Trauma

Maltreatment histories will be measured with the Childhood Trauma Questionnaire – Short Form (CTQ-SF), a 27-item 5-point Likert-type self-report questionnaire [97]. The CTQ-SF consists of five subscales: physical abuse, sexual abuse, emotional abuse, physical neglect and emotional neglect. The questionnaire has demonstrated high [98] to acceptable [99] internal consistency, good convergent and discriminant validity [98] and good criterion-related validity [97]. The CTQ-SF will be administered at baseline.

#### Attachment style

The Attachment Styles Questionnaire (ASQ), a 24-item 5-point Likert-type self-report questionnaire [100] is used to generate individual measures of four attachment styles: secure, fearful, dismissive and preoccupied. The construct validity of the four scales is adequate as well as the reliability, except for the dismissing style [101]. The ASQ will be administered at baseline and 12 months after baseline.

#### Demographic characteristics

Demographic and general patient characteristics, such as treatment history, criminal history, medication use, age, gender, ethnicity, educational level and marital status, will be collected. A self-developed questionnaire will be administered at baseline, 12 months and 18 months after baseline.

#### Working alliance

The Working Alliance Inventory - short form (WAI-sf) [102, 103] a 12-item 5-point Likert-type self-report questionnaire, will be assessed to measure the therapeutic alliance of patient-therapist pairs. The WAI-sf measures three domains of therapeutic alliance: 1) patient-therapist agreement on the (value of) treatment goals, 2) patient-therapist agreement about the tasks (to achieve goals), and 2) the quality of the bond between patient and therapist. Psychometric properties are found to be adequate [102, 104]. The questionnaire will be filled out by both patient and coach at 3, 6, 9 and 12 months after baseline.

#### Intellectual disabilities

The Screener for Intelligence and Learning Disabilities (SCIL) is administered at baseline if official, recent information (not older than 2 years) about IQ levels of the patient is not available. The SCIL is a Dutch screening instrument that is validated and often used within similar populations to detect IQ levels under 85 [105]. The instrument consists of multiple assignments such as calculating, writing, reading, spelling, understanding of a proverb and drawing. Furthermore, the total score is also based on questions about the education level, use of intellectual disability services, reading behavior and the availability of family support. The SCIL demonstrated adequate sensitivity and specificity [105].

#### Cognitive distortions

Cognitive distortions will be measured at baseline with de How I Think Questionnaire (HIT-Q), a 54-item 6-point Likert-type self-report questionnaire [106–108]. Of these items, 39 can be clustered into four categories of self-serving cognitive distortions: 1) self-centeredness, 2) blaming others, 3) minimizing/mislabeling, and 4) assuming the worst. All beforementioned 39 items are matched to one of the four antisocial behavioral categories of the DSM-IV-TR: 1) opposition-defiance, 2) physical aggression, 3) lying, and 4) stealing. Furthermore, other items consist of anomalous-responding items (8 items) and positive fillers (7 items). The HIT-Q demonstrated good psychometric properties in terms of reliability as well as convergent and divergent validity [109, 110].

#### Program integrity

In order to assess whether the intervention is implemented as intended and in accordance with protocols, program integrity is measured with a self-developed questionnaire. The instrument consists of questions about general characteristics of coaches, program evaluation and an estimation of patients’ adherence or motivation. Researchers will fill out the questionnaires together with coaches at the end of the intervention (12 months after baseline).

### Other variables of interest

A qualitative interview of experiences will be obtained with self-developed topic lists [111] to evaluate experiences of both participants and network coaches during coaching.

### Ethical and safety reporting

All Adverse Events (AE; e.g. undesirable events that occur to a subject during the study) and Serious Adverse Events (SAE; e.g. life-threatening medical events resulting in significant disabilities, hospitalisation or death) will be documented by the researchers. The principle investigator and ethics committee will be informed when a SAE occurs.

### Data management

After inclusion, a unique project number will be allocated to each participant. The key of these project numbers will be stored in a password protected file. All paper and digital data will be stored encoded in an online validated data management environment - Castor EDC - by a research assistant. All stored data will be double checked, corrected (if necessary) and signed. Only principal investigators and trained research associates will have access to Castor EDC and research files. Paper questionnaires, other data and informed consents will be stored in a locked cabinet at the participating site. Data will be stored for 15 years according to legislation. Storage of data will be supervised by the principal investigator in compliance with the Dutch Personal Data Protection Act.

The study will be conducted based on the quality handbook of the Amsterdam Public Health (APH) research institute. Data monitoring is provided by independent researchers at the Arkin Institute for Mental Health. An audit can be initiated by an independent researcher of the Amsterdam University Medical Centre throughout the course of the study. During an audit the independent researcher will investigate whether the execution of the study is based on the quality standards of the APH research institute.

## Statistical analysis

An intention-to-treat analysis will be included in the data analyses of the primary outcome measure (mental wellbeing) and secondary outcome measures (psychiatric functioning, criminal recidivism, substance abuse, quality of life, social network, social support, loneliness and self-sufficiency). Besides, subgroup analyses will be conducted on the protocol completers sample. Generalized Linear Mixed Models (GLMM) in Statistical Package for the Social Sciences (SPSS) will be used to model all continuous outcome measures, considering the distributional characteristics of the data. If the normal distribution assumption is violated, resampling methods will be used to transform data into a normal distribution or appropriate non-parametric tests will be used. Generalized Estimating Equations (GEE) will be used to analyze the categorical outcome measures. The main between-group effect (TAU versus FNC) and the interaction effect (group * time) will be analyzed whilst correcting for baseline assessment. Outcome data of all assessments will be included in the analyses to examine the differences in the development in time between both groups. The criminal recidivism data will be analyzed using Survival Analysis and corrected for time at risk. Missing data will be addressed using multiple imputation or statistical techniques that control for missingness-at-random.

To assess the magnitude of treatment effects on the primary outcome and secondary outcomes, Cohen’s *f* effect size will be calculated. Effect sizes of *f* = 0.40 are considered large, effect sizes of *f* = 0.25 are considered moderate and effect sizes of *f* = 0.10 are considered small (Cohen, 1988).

Moderator analyses will be used to examine potential moderators of treatment outcome by adding potential moderators as covariates of factors to the GLMM/GEE analyses. Furthermore, multilevel mediation models will be carried out to examine potential mediators of treatment outcome. At last, appropriate statistical methodology in ATLAS.ti will be used for the analyses of the qualitative data [111].

## Discussion

This article describes the study protocol of a randomized controlled trial to examine the effectiveness of the addition of an informal social network intervention (FNC) to TAU in a forensic psychiatric outpatient population. Considering the severe and multiple problems that are observed in forensic populations, more knowledge about multimodal evidence-based interventions is warranted. Social network-related problems of forensic psychiatric patients are considered one of the important targets in forensic treatment. Previous research indicates that the absence of a supportive social network can increase the risk of criminal recidivism, whereas a supportive social network can decrease the risk of criminal recidivism. Despite this, evidence-based interventions aimed at improving social networks of forensic outpatients are not available. Therefore, this RCT examines the effectiveness of the addition of FNC to TAU in improving mental wellbeing, psychiatric functioning and decreasing criminal recidivism of forensic psychiatric outpatients. We will also examine the effectiveness of the addition of FNC to TAU on several secondary outcome measures. Furthermore, this study will explore the development of a broad range of primary and secondary outcomes over time and the impact of relevant patient- and intervention-related characteristics. If effectiveness is shown, the social network intervention - based on a collaboration between formal and informal care - can be implemented more extensively within the field of forensic mental health care.

The present study has several strengths. First, to our knowledge, this is the first RCT that examines the effectiveness of a social network intervention within a forensic outpatient population. The field of forensic (outpatient) care is in urgent need of more RCTs in order to gain knowledge about evidence-based practice [23, 24]. Therefore, this study will contribute to the existing knowledge about effective forensic interventions. Secondly, this RCT is embedded in clinical day-to-day practice resulting in high ecological study validity. Hence, it is possible to account for the specific needs of patients, professionals, managers and policy makers. Thirdly, the research group has chosen to include a wide range of primary and secondary outcome variables. Multiple participant and intervention characteristics will be examined to provide insight regarding potential mediators and moderators of treatment effectiveness. Besides, many different types of instruments (i.e. electronic patient files, self-report instruments, reports from coaches and registrations from trained researchers and information sources) will be used to gather this information. Information about program integrity and dosage will be studied as a potential moderator of intervention effects. Moreover, qualitative and quantitative methods will be combined to study intervention effects. Lastly, the large amount of clinically relevant outcome measures will be administered during a long follow-up period of 18 months with multiple assessment points. Therefore, it will be possible to gather valuable information about changes in important life areas over a longer period of time.

Given the beforementioned strengths, several pragmatic challenges are considered important. In our opinion, the first challenge of this study will be obtaining sufficient patients for inclusion in the study. Our study sample consists of complex and vulnerable patients with problems in multiple areas of life. Many of them have long mental health care histories and extensive criminal records. Former negative experiences with care or criminal justice services may have affected their attitude towards formal care and affiliated scientific research projects. Therefore, an additional social network intervention may not yet be indicated by care professionals during TAU or difficult to implement. Moreover, the majority of patients are obligated to participate in forensic treatment. Patient’s motivation for participation in a prolonged scientific research project and an additional intervention might therefore be low. Subsequently, the execution of a long follow-up period with multiple assessments in everyday clinical practice is expected to be challenging. In order to address these issues in inclusion and data collection, researchers will collaborate with mental health care professionals as much as possible considering the ethical and privacy guidelines. The researchers will participate in general team meetings. The team of researchers will be trained to keep an assertive, outreaching and flexible attitude towards both professionals and patients. Current contact information of the patient and his/her social network members is gathered actively during all follow-up assessments. Patients receive an appropriate financial compensation to emphasize our appreciation for their contribution and time-investment. Besides, researchers will put effort in building and maintaining contact with patients through phone and e-mail. When researches fail to reconnect with patients, network members will be contacted and home visits will be planned. A final important concern is the risk of nonadherence to intervention and treatment drop-out. To account for this risk, postponing the moment of randomization was considered. Unfortunately, the characteristics of the social network intervention do not allow this adjustment since the referral process (i.e. selection, intake and matching) of the intervention is complicated and time-consuming. Circumstances of patients might change during the referral period, therefore randomization must be performed as soon as possible after inclusion. Close cooperation with informal care will be used to enlarge the accessibility and attractiveness of the social network intervention. During the course of the study, at follow-up assessments, patients will be actively encouraged to participate in the intervention by researchers. Besides, when patients tend to drop-out professionals will be asked to motivate patients. Patients who drop-out during the study will still be included in the follow-up measures which enables us to examine profiles of treatment success and failure.

Finally, several methodological limitations should be considered. In order to promote generalizability of data and to examine which patients might benefit from the intervention, researchers chose to make the study accessible for a wide range of forensic patients. Consequently, this leads to a heterogeneous study sample which makes it difficult to achieve high internal validity. Furthermore, many changes in important areas of life, such as hospitalisations, relapses in substance (ab) use or criminal behaviour and changes in work, living environments and close relationships are expected to occur regularly during the study. These changes might influence the intervention and outcome measures. To account for these confounding factors, multiple participant and intervention characteristics will be recorded throughout the course of the study. Besides, the RCT design enables controlling for confounding factors between groups.

## Conclusion

This study will examine the effectiveness of the addition of an informal social network intervention to treatment as usual with a RCT design. It is expected to contribute to the development of evidence-based interventions for (outpatient) forensic psychiatric populations. If effectiveness is shown, forensic mental healthcare could be optimized by collaborating with informal care or other community initiatives.

## Data Availability

The datasets used and/or analysed during the current study are available from the corresponding author on reasonable request. All authors will have access to the complete dataset.

## Abbreviations

ACT: Assertive community treatment
AE: Adverse Events
APH: Amsterdam Public Health
ASQ: Attachment Styles Questionnaire
CBT: Cognitive behavior therapy
CTQ-SF: Childhood Trauma Questionnaire – Short Form
EDC: Electronic Data Capture
DSM-IV-TR/5: Diagnostic and Statistical Manual of Mental Disorders – Fourth edition, Text Revision / Fifth edition
EMDR: Eye Movement Desensitization and Reprocessing
FACT: Flexible / function assertive community treatment
FNC: Forensic Network Coaching
GEE: Generalized Estimating Equations
GLM: Good Lives Model
GLMM: Generalized Linear Mixed Models
HIT-Q: How I Think Questionnaire
HoNOS: Health of the Nations Outcome Scales
IPOL: International Police Information Service
JDS: Justice Documentation System
MANSA: Manchester Short Assessment of Quality of Life
MATE 2.1: Measurement in the Addictions for Triage and Evaluation
MHC-SF: Mental Health Continuum - Short Form
NGI: Name Generator/Interpreter method
MINI: Mini-International Neuropsychiatric Interview
RCT: Randomized controlled trial
RNR: Risk Need Responsivity
SAE: Serious Adverse Events
SCIL: screener for learning disabilities and intelligence
SPSS: Statistical Package for the Social Sciences
SRD: Self-Reported Delinquency scale
SSL: Social Support List
SSM-D: Dutch Self-Sufficiency Matrix
TAU: Treatment as usual
UMC: University Medical Center
WAI-sf: Working Alliance Inventory - short form

## Footnotes

1 De Regenboog Groep is an independent informal care foundation based in Amsterdam, the Netherlands.
